# Integrated *-Omics*: A Powerful Approach to Understanding the Heterogeneous Lignification of Fibre Crops

**DOI:** 10.3390/ijms140610958

**Published:** 2013-05-24

**Authors:** Guerriero Gea, Sergeant Kjell, Hausman Jean-François

**Affiliations:** Department Environment and Agro-biotechnologies (EVA), Centre de Recherche Public-Gabriel Lippmann, 41, Rue du Brill, L-4422 Belvaux, Luxembourg; E-Mails: guerrier@lippmann.lu (G.G.); sergeant@lippmann.lu (S.K.)

**Keywords:** systems biology, *-omics*, fibre crops, lignin, bast fibres, secondary cell wall

## Abstract

Lignin and cellulose represent the two main components of plant secondary walls and the most abundant polymers on Earth. Quantitatively one of the principal products of the phenylpropanoid pathway, lignin confers high mechanical strength and hydrophobicity to plant walls, thus enabling erect growth and high-pressure water transport in the vessels. Lignin is characterized by a high natural heterogeneity in its composition and abundance in plant secondary cell walls, even in the different tissues of the same plant. A typical example is the stem of fibre crops, which shows a lignified core enveloped by a cellulosic, lignin-poor cortex. Despite the great value of fibre crops for humanity, however, still little is known on the mechanisms controlling their cell wall biogenesis, and particularly, what regulates their spatially-defined lignification pattern. Given the chemical complexity and the heterogeneous composition of fibre crops’ secondary walls, only the use of multidisciplinary approaches can convey an integrated picture and provide exhaustive information covering different levels of biological complexity. The present review highlights the importance of combining high throughput *-omics* approaches to get a complete understanding of the factors regulating the lignification heterogeneity typical of fibre crops.

## 1. Introduction

In the list of highly sought-after goals for a sustainable exploitation of natural resources, the use of plant-based material to replace fossil carbon-derived products ranks undoubtedly among the top. The increase in the world population, together with rapid industrial development, push towards the depletion of petrochemicals and the subsequent need of finding sustainable sources of raw materials. Plant-sourced raw material, *i.e.*, lignocellulosic biomass, is such a renewable resource that can reduce our dependence on fossil carbon. Plant cell walls are indeed valuable natural products which find a wide array of industrial applications, spanning from conversion into energy to material development. The food-oriented agricultural industry is progressively being redirected towards one ensuring also raw material for pulp and paper, textiles, construction and biofuels [1–3]. This aspect is particularly important, as it offers an alternative to forest-sourced biomass. In view of the foreseen deficit in wood caused by the decreasing capacity of the world’s forest to supply woody biomass [4,5], much effort is devoted towards finding other sources of renewable raw material. The use of non-woody plants, as well as agricultural byproducts (e.g., corn, wheat, rice, sorghum, barley, sugarcane, pineapple, banana, coconut [6]), which can supply lignocellulosic biomass for different industrial needs, is therefore receiving increasing scientific and societal interest. The advantages of non-woody plants are clear: short growth cycles, moderate irrigation requirements, relatively low lignin content [1,3] are emblematic examples. Although agricultural byproducts such as rice husks can be used for the production of energy, the consistent quality required for most industrial processes can only be met by the cultivation of plants specifically grown for fibres, collectively known as fibre crops. These are non-woody fibres, which hold great potential as sources of raw materials for different industrial sectors. A prime example of the impact of fibre composition on the usability of plant biomass is offered by alfalfa (*Medicago sativa*), that has been selected for centuries for a decreasing lignin-content, to increase the digestibility of this queen of forages.

Fibre crops comprise flax (*Linum usitatissimum* L.), hemp (*Cannabis sativa* L.), ramie (*Boehmeria nivea* L.), jute (*Corchorus capsularis* L. and *Corchorus olitorius* L.), kenaf (*Hibiscus cannabinus* L.), sisal (*Agave sisalana* Perr.). Other plants that are of much less importance on a global scale, but are nonetheless cultivated for their fibres, include nettle (*Urtica* spp. [7]), okra (*Abelmoschus esculentus* [8]), banana (or abaca, *Musa textilis*). The most important fibre crop, when looking at global annual production, is without any doubt cotton; however cotton fibres are enlarged trichomes and thus have an origin completely different from the fibres discussed in this review.

The fibrous mass of these plants is characterized by a low lignin content and by the occurrence of fibres highly enriched in cellulose. These features make them extremely appealing not only as models to study secondary cell wall formation, but also as sources of valuable raw materials used in different industrial sectors. Their fibres (called bast fibres) show physical properties (namely tensile strength) which make them optimal for the woven textile industry, but at the same time their low lignin content is a desirable feature for biomass saccharification. Their woody core fibres (a.k.a hurd or shives) are excellent for paper production, as insulating material, as fibrous component in concrete and wall coatings and once crushed to powder they can be used to make biodegradable plastics. It is hence clear that progressing in an integrated study of the mechanisms which regulate the tissue-specific secondary cell wall composition of fibre crops can help devise strategies to maximize the exploitation of their lignocellulosic biomass.

## 2. Plant Fibres: Nature’s Treasure Trove

Plant lignocellulosic fibres hold great value both for the environment and the economy, as they favor the shift towards a bio-based economy. Moreover, they shape important plant features, as the mechanical properties of wood and the quality of textiles and paper. True fibres are cells of the sclerenchyma, *i.e.*, plants’ mechanical tissue [9], characterized by the presence of secondary cell walls. Several aspects of fibre ontogenesis are still not understood [10,11]; however studies on model plants have proved very useful to shed light on some mechanisms. It is here worth mentioning the work carried out on *Arabidopsis thaliana* which has delivered milestone data concerning, for instance, the cascade regulating the transcriptional wiring of secondary cell wall formation [12–26]. Moreover, *A. thaliana* offers a wide collection of cell wall mutants’ [27–37] and by modulating its growth conditions (as photoperiod, temperature, hormone treatment [10]), it is possible to affect fibre formation. It was furthermore demonstrated that this herbaceous model plant can be a useful system for the study of gelatinous fibres’ (G-fibres) formation [38]. The limits in the study of fibre formation encountered with *Arabidopsis* can be overcome with poplar, which has contributed to gain a deep understanding on the formation of woody fibres in trees [39–45]. The availability of the genome [46], together with the possibility of downregulating gene expression [47], makes poplar an attractive model for fibre secondary cell wall formation. However, a detailed study on isolated fibres is challenging, because they are usually found mixed with other cell types [11], and therefore a preparation of pure poplar fibres is very difficult to attain. Important data concerning secondary cell wall biogenesis come also from the legume model plant *M. truncatula* [48–51], which was indeed shown to be an appropriate system for cell wall studies.

**Figure 1:**
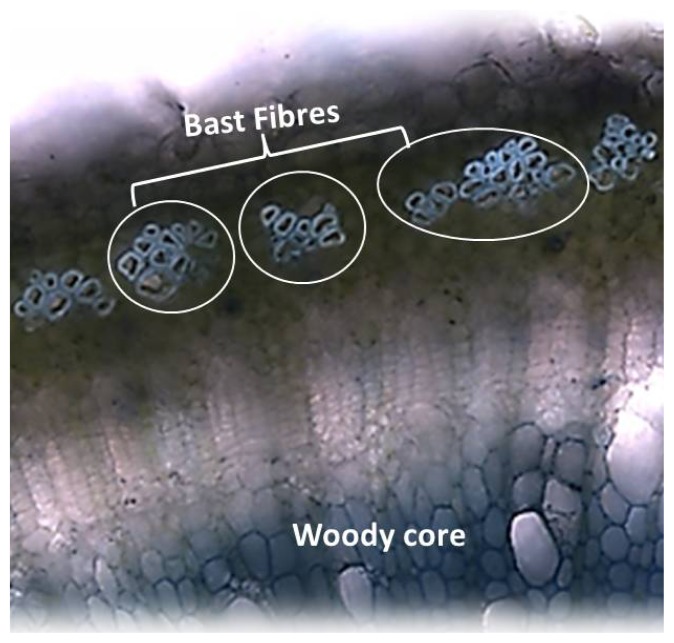
Cross section of *C. sativa* stem (Toluidine Blue staining), showing the bast fibres surrounding the woody core.

Fibre crops, principally hemp and flax for which the genomes are available [52,53], are excellent systems for the analysis of fibre formation and the great advantage they offer is the possibility of studying tissues with dramatically different lignification patterns in the same plant. The stem of these fibre crops shows a distinct anatomical structure which makes them particularly suitable for molecular studies. Their cortical bast fibres, characterized by the occurrence of a high percentage of cellulose and a low lignin content, envelop a central lignified core (Figure 1), a radial distribution that favors the study of tissues with contrasting composition that are isogenic and grown under exactly identical conditions. Therefore the application of high throughput holistic (proteomics, metabolomics, transcriptomics), as well as targeted approaches (expression analysis of a subset of genes, targeted metabolite profiling), is feasible. The outer tissue can be peeled off from the woody core and the isolated material is devoid of “contamination” coming from the lignified inner tissue.

The use of an integrated biology approach can help to shed light on the molecular processes which intervene in the two tissues and determine the contrasting nature of secondary cell walls’ composition. Although some molecular studies (mainly concerning flax and hemp tissues) are available in literature (summarized in Table 1), a systems biology approach integrating multilevel *-omics* data is still missing. In the last decade the approach to biological studies has changed and these are now conducted at the systems level [54,55], through the integration of data from different sources (*i.e.*, integrative systems biology). The integration of all these data flows into a general model which, applied to fibre crops, can provide a depth of analysis never so far attained.

## 3. Lignin: The Lord of the (Aromatic) Rings

Plant secondary metabolism comprises an array of complex pathways which shape the biochemical plasticity of plants. Considered dispensable for plant survival, it fuels cells with important organic compounds which mediate relevant events, as for instance defense and structural support. Within plant secondary metabolism, the phenylpropanoid pathway undoubtedly occupies a central position, as it generates the common precursor, *p*-coumaryl CoA, for the biosynthesis of flavonoids and lignin building blocks. Molecules from this pathway are involved in structural support (lignin), redox homeostasis (chlorogenic acid), reproduction (flavonoids as flower pigments) and many other aspects of plant biology [56,57]. The importance of this metabolic route is evidenced by numerous recent studies aimed at increasing our understanding of this pathway and often related to redirecting cell wall lignification through genetic manipulation [58–63]. The main goal of such studies was to increase the digestibility of plant biomass favoring bio-ethanol production, either through a transgenic approach [64–71], or through the incorporation of monolignol analogs/substitutes [72–74].

Lignin, a polyaromatic, hydrophobic, heterogeneous biopolymer, is the main product of plant secondary metabolism, and is in mass globally produced second only after cellulose. Its structural complexity confers an intrinsic “chaotic” nature and often theoretical analyses spanning from fractal geometry to deterministic chaos concepts are invoked to describe the multi-level intricacy of its organization [91]. This surprising structural complexity constitutes a sort of chemical “fingerprint”: It might be virtually impossible to find in nature two identical lignin macromolecules with the same degree of polymerization or succession of phenylpropane units, and therefore it is often preferred to refer to this aromatic polymer as “lignins” [91]. Recently, improvements to techniques such as atomic force microscopy, FTIR spectroscopy [92], and NMR [93] have greatly helped in fostering the structural study of lignin.

Lignin constitutes an inspiring model for the creation of biomimetic materials with desirable physical properties and this is an issue of particular relevance for the construction sector, which is shifting towards an eco-friendly building concept, through the use of plant-sourced materials instead of petrochemicals. Lignin contributes indeed to the elastic modulus of timber while conferring strength, and is water-proof.

The physiological role of lignin is that of biological “glue” for wall polysaccharides, and its presence impairs saccharification of plant biomass, as it impregnates cellulose, thus hindering its accessibility to hydrolytic enzymes. However, its function is to ensure vascular integrity, fortify the cell wall against pathogen attack and to confer increased hydrophobicity and mechanical rigidity.

Plant secondary cell walls show a sort of “promiscuity” towards lignin structures: They can tolerate variation in the polymer macrostructure, therefore providing extreme flexibility in situations of environmental stress or in case of transgene-induced modifications. The enzymes which intervene in the lignin biosynthetic pathway, the efficiency in energy and carbon retention of which were recently calculated [94], constitute a metabolic “grid” [95,96] which ensures plasticity in case of natural or artificial modifications. Multigene families indeed exist [97–101] and isozymes can function in case of alterations in the pathway [96].

Metabolic channeling in the phenylpropanoid pathway has been demonstrated for phenylalanine ammonia-lyase (PAL) and cinnamate-4-hydroxylase (C4H) [102,103] and recent evidence in literature has shown the existence of protein-protein/protein-membrane interactions for CYP73A5 and CYP98A3, two *Arabidopsis* cytochrome P450 catalyzing the hydroxylation of the phenolic ring of monolignols [104]. The enzymes involved in the pathway seem therefore to present physical cross-talk which fine-tunes the pathway.

Although the availability of different plant mutants defective in genes involved in the lignin deposition route [105–107] has contributed to shed light on some of its biosynthetic aspects, certain events, as for instance polymerization and precursors’ export, are only starting to be tackled [108,109]. For a recent review on the knowledge on lignin deposition and the gaps in the latter see Liu [110]. The analysis of plants with altered lignification by means of an integrated approach can provide valuable data and can fill the gaps still present in the study of secondary cell wall formation in plants.

## 4. The Particular Cell Wall of Bast Fibres

A great heterogeneity in wall lignification exists in nature: Among different species, in the different tissues within the same plant [111] and in response to biotic/abiotic stresses [96,111,112]. Particularly rich in lignin heterogeneity are plant fibres. One of the most striking examples of differential lignification (both qualitative and quantitative) within the same plant is found in fibre crops. These plants contain small amounts of lignin, which is abundant in the core, while the cortex harbors bundles of bast fibres particularly rich in cellulose.

Bast fibres are cortical extraxylary sclerenchymatous structures which provide mechanical support to the conductive elements of the phloem and are typically present in bundles held together by pectins and lignin. Their cell walls are composed primarily of cellulose (which can make up 75%–80% of the dry mass in *C. sativa*), hemicelluloses (5%–16%) and pectin (1%–4%) [113], while lignin is a minor component (2%–7%), associated to the middle lamella. Some fibre crops, like hemp, display the presence of both primary and secondary bast fibers: Primary fibres are procambium-derived long (from 20 to more than 100 mm of length) and strong fibres containing little lignin, while secondary fibres are shorter (around 2 mm in length), more lignified and derive from the cambium. From the cellular point of view, bast fibres are unique, since they are characterized by the occurrence of a gelatinous type of secondary cell wall (the so called G-layer), which is rich in crystalline cellulose with low microfibril angle [114,115]. For a summary of the main physical parameters of bast fibres, see Table 2.

G-layers are usually found in reaction wood (*i.e.*, tension wood) in response to a mechanical stimulus and can generate high tensional stress. However bast fibres do not play the same contractile role as tension wood [117,118] and are characterized by the occurrence of a specific type of β-1,4-galactan [119]. The cell wall chemistry, together with their mechanical features, make bast fibres highly attractive and valuable for the development of biomimetic material and for use as feedstocks with desirable improved saccharification traits [120].

The formation of bast fibres takes place through intrusive growth, a mechanism which increases the number of fibres in cross sections, while keeping their total number unchanged within a specific stem segment [121]. The mechanism of primary fibres’ intrusive growth has been studied in more detail than that of secondary fibres and it was shown that the procambium close to the apical meristem is the starting site of primary fibres’ elongation, which in flax was proposed to occur through diffused growth [11,121–123]. Studies on flax bast fibres have shown the occurrence of a galactan-rich layer (Gn-layer) which is progressively modified through the activity of β-galactosidase into the cellulosic G-layer [83]. Moreover the process of G-layer formation in flax is preceded by the accumulation of “bicolor” Golgi vesicles which fuse forming large vacuoles [124,125]. These vacuoles fuse then with the plasma membrane and release their cargo in a “syringe-like” fashion; this mechanism ensures the maximal delivery of the vesicles containing matrix polymers of the Gn layer to sites where cellulose is present, when the cell wall is still in its construction phase [125]. The stem of fibre crops shows not only a radial distribution of lignification pattern (the lignification degree increases as we move centripetally in the stem), but also a longitudinal distribution of cell wall metabolic stages. Along the axis of flax, it was demonstrated that a physically distinctive point is present, called the “snap point” [122] which is a zone where the mechanical properties of fibre cells change dramatically. The region above the snap point is characterized by cell elongation through intrusive growth, while below it cell wall thickening takes place. Below the snap point cell walls show a typical bipartite appearance [83,122,124]: A layer with loosely-packed structure (that is the Gn-layer) and one more ordered and homogeneous (the G-layer). At maturity the Gn-layer is remodeled into a G-layer. Interestingly, the lignin present in flax and hemp bast fibres is condensed [3,76,78], with a high occurrence of G and H units (H units are 5% in the core and 13% in flax bast fibres). This characteristic, together with the high crystallinity of cellulose, might have consequences on the digestibility of fibre crops’ cellulose, which, although only enrobed by a low amount of lignin, is extremely recalcitrant to saccharification.

It was recently shown that the typical hypolignification of flax stem is accompanied by the high accumulation of phenolics compounds and by a very active monolignol metabolism [86]. Using lignomics, 81 different phenolic compounds were identified in the outer stem tissues, 65 of which were reported for the first time in flax. These results show the complexity of phenylpropanoid metabolism in flax stems (and most likely in other fibre crops), which is transcriptionally regulated and translates into a range of organic compounds belonging to intricated metabolic routes which are still not fully unveiled.

## 5. Integrated *-Omics* to Study Differential Lignification Patterns in Fibre Crops

Secondary cell wall biosynthesis is a good example of a complex physiological system, as it involves the coordinated expression of cellular activities at different biological levels (*i.e.*, transcriptional, protein- and metabolite-level). High throughput analyses can deliver a wealth of data, but generally only convey partial information [126]. In this post-genomic era, where the amazing progress in sequencing allows us to reach resolutions and speed never before attained, the study of organisms has moved towards an integrated vision, which tries to explain cellular responses to natural or induced perturbations (*i.e.*, a certain phenotype) by combining high throughput *-omics* data [127]. This shift towards systems biology is witnessed by the progressively increasing number of bioinformatics tools/portals enabling the integration of *-omics* data (e.g., Paintomics [128]; MADMAX, [129]; BESC, [130]; COVAIN [131]). The availability of microarray and metabolomics data allows not only the *in silico* analysis of gene co-expression networks, but also of gene-to-metabolite connections [126]. This meta-analysis of *-omics* data can be further extended to genomics and proteomics, thus enabling for instance metabotyping at the ecotype-level [126,132]. DoE (design of experiments) and multivariate analysis can then be applied to systems biology to better interpret the data and deduce models [133].

Only a handful of studies are available in literature on fibre crops’ transcriptomics (summarized in Table 1). This is probably due to the lack of genomes for many fibre crops, which makes bioinformatics analysis more challenging, although molecular studies aimed at amplifying and studying genes involved in lignin and cellulose biosynthesis from fibre crops are present in literature [134–136]. The majority of the transcriptomics data come from microarray and cDNA libraries studies on flax and hemp aiming at understanding the molecular events behind cell wall dynamics in stem/hypocotyls tissues [77,79,81,83–86]. These studies have shown how genes involved in cell wall biosynthesis and remodelling display typical expression patterns which mark specific stages of development in the different stem tissues and along different regions of the stem axis. Hypolignification was indeed demonstrated to be associated with the low abundance of monolignol biosynthetic gene transcripts, laccases and some peroxidases in stem outer tissues and to the occurrence of transcription factors involved in lignin biosynthetic pathway repression (*i.e.*, orthologs of AtMYB4, EgMYB1, ZmMYB31/42 [86]). The establishment of the parallelism between sequential stages of hypocotyl development (namely 7, 9 and 15 days) and the differentiation of bast fibres along the top, middle and bottom region of the stem [83], has offered a supplementary tool in the study of phloem fibre development. It was indeed demonstrated that flax phloem fibres development is strictly synchronized with hypocotyls elongation and that the hypocotyl and stem show a set of common genes activated during their specific developmental/differentiation stages. Elongating hypocotyls showed enrichment in transcripts involved in photosynthesis, transport, hormone signaling, as well as primary cell wall deposition [83]. Arabinogalactan proteins (AGPs), β-galactosidase were enriched in the transition between elongation and secondary wall deposition, while chitinases and glycosyhydrolases (GHs) like KORRIGAN were abundant at later stages [83].

The availability of next generation sequencing (NGS) techniques is now only starting to be applied to fibre crops to perform for instance genome-wide SNPs discovery (which can facilitate (Quantitative Trait Loci) QTL identification [137]) and to carry out *de novo* transcriptome assembly to identify crucial cell wall-related genes, as cellulose synthases (*CesA*s) [90]. Being a sequencing-based technology, NGS can reach accuracies which go beyond the limits imposed by the microarray technology. It widens the limits of detection to low-abundant transcripts and no previous knowledge of genomes is necessary, as it can be used to create sequence assemblies, to map reads, exon/intron boundaries, splicing variants [138]. NGS technologies are also finding fertile fields in the study of microRNA (miRNA and the so-called “miRNomics”). Increasing evidence on the role of miRNAs in the regulation of cell wall metabolism do exist in literature [87,139,140], and they constitute very interesting targets for engineering plants secondary cell walls to meet industrial and bioenergetic needs [139]. In poplar, for instance, the presence of unique miRNAs (miRX41) targeting a cellulose-synthase like gene, as well as NAC transcription factors and a xyloglucan endotransglycosylase/hydrolase (XTH16) (miRX50), have been recently identified through SOLiD ABI sequencing technology [139]. A previous study showed also that poplar-specific miRNAs (not present in *Arabidopsis*) responsive to mechanical stress exist, and that they are associated with the biosynthesis of cell wall metabolites [141]. The sensitivity of NGS applied to the mining of miRNAs involved in cell wall metabolism in fibre crops’ stems can help identify novel candidates targeting transcription factors and/or genes involved in the heterogeneous lignification pattern observed.

As for transcript and metabolite profiling, most work on the proteome abundance in fibre crops has been focused on flax. Recently Day *et al.* reported the identification of 152 predicted cell wall proteins in flax stems using a sequential salt extraction [89]. The majority of cell wall proteins is involved in sugar and/or glycoprotein-related events and includes numerous glycosidases but also chitinases [89]. Of the proteins known to be involved in lignin deposition and thus secondary metabolism, several class III peroxidases were identified [142,143], the gene expression of which was previously shown to be different between outer- and inner- (hypolignified) flax stem tissues [88]. One of these peroxidases was previously identified as being a cell wall protein in another study on flax [86].

While the previous studies isolated the different tissues or compared the proteome of the stems at different developmental stages, the most targeted study on fibre proteome was reported by Hotte and Deyholos [82]. In this study DIGE was used to compare the protein abundance in isolated fibres *versus* the surrounding cortical tissue. From the 240 spots that were more intensely stained in the fibres, corresponding to enriched proteins, 51 were identified [82]. This study indicates that fibres are, as expected, devoid of proteins related to photosynthesis and enriched in proteins involved in cell wall deposition. Of the 10 proteins with the highest enrichment factor, 7 are directly involved in cell wall polysaccharide metabolism with the most important one being β-galactosidase. Another enzyme involved in cell wall polysaccharide metabolism is rhamnose biosynthetic enzyme. Fructokinase was also identified in several spots that were more abundant in the fibres. Given the low lignin content in early stage developing flax fibres, just below the snap point, it is not surprising that no enzymes related to phenylpropanoid metabolism were found. However the enrichment of a secretory peroxidase might indicate the start of lignin deposition.

Although proteomics has proven its power to contribute to the elucidation of pathways activated during cell wall development, the enzymes responsible for the biosynthesis of secondary metabolites, mainly the phenylpropanoid pathway with monolignols as major product, are of low abundance and therefore generally not detected. Furthermore key steps in phenylpropanoid metabolism are catalyzed by cytochrome P450-like membrane-anchored proteins [144,145], making dedicated approaches necessary for their proteome-level study.

## 6. Conclusions and Future Perspectives

The stem of fibre crops represents one of the best examples in nature of contrasting lignification pattern existing within the same plant. This heterogeneous secondary cell wall composition allows the use of fibre crops’ lignocellulosic biomass for different industrial sectors. Although recent studies have contributed to shed light on some aspects controlling the different lignification profile in these plants, an integrative study obtained by simultaneously interrogating the organisms at different levels of biological complexity is still missing. To fully understand the molecular events responsible for secondary cell wall biogenesis in fibre crops, it will be necessary to integrate high throughput *-omics* analyses with accurate experimental design, bioinformatic tools for meta-analysis and co-visualization of the data and mathematically-supported models inference. The integrated knowledge thus obtained can be applied to test the models through functional genomics: Modifications of cell wall metabolic networks can be engineered and used to improve the extractability of fibres (*i.e.*, retting), the agronomic traits of existing cultivars, or to create application-specific fibres.

## Figures and Tables

**Table 1:** Summary of the available studies on fibre crops addressing transcriptomics, metabolomics or proteomics.

Species	Type of study	Tissue(s)	Relevant results	Reference
***L. usitatissimum***	histology, GC-analysis	inner and outer stem tissues	different pattern of laccase-gold particles detected in stem tissues which relates to phenolic compounds	[75]
***L. usitatissimum***	histochemistry/chemical analysis	bast fibres at 2 developmental stages	xylem and bast fibres rich in G units, H units present in bast fibres	[76]
***L. usitatissimum***	ESTs from cDNA library/RT-PCR	outer stem tissue	highly expressed cell wall-related ESTs found: *CesA*s, XTH, peroxidase, β-xylosidase and β-galactosidase	[77]
***C. sativa***	microscopy/chemical analysis	bast fibres from apical and basal regions of the stem	condensed lignin in stem, *p-*hydroxyphenyl units decline with maturity	[78]
***C. sativa***	cDNA microarray/qPCR	outer stem tissue top, middle, bottom	GT transcripts enriched in the middle and bottom regions, GH transcripts enriched in the top region; top region rich in PRPs; middle and bottom regions rich in AGPs	[79]
***L. usitatissimum***	cDNA library	fibre-bearing phloem tissues from stem/3 segments along stem axis	AGP, LTP enriched in elongation and cell wall-thickening regions; chitinases, β-galactosidases enriched also in specific stages of stem development	[80]
***C. sativa***	cDNA microarray/lignin histochemistry/chemical analysis/Northern blot	wooden core *vs.* bast fibres in the top, middle, bottom regions	upregulation of PP and shikimate pathways, AAA and lignin biosynthesis, C1 metabolism in the core tissue; AGPs, genes in lipid/wax metabolism and photosynthesis upregulated in bast tissues	[81]
***L. usitatissimum***	histology, 2D-DIGE	isolated fibres, cortical tissue	enrichment of β-galactosidase, rhamnose biosynthetic enzyme	[82]
***L. usitatissimum***	cDNA microarray/qPCR/activity staining	hypocotyls at 7, 9 and 15 days	genes involved in primary wall deposition upregulated in the early stage, AGPS and β-galactosidase expressed in the middle stage, secondary metabolism and GH transcripts upregulated in the last stage	[83]
***L. usitatissimum***	454 sequencing of high-density oligo-microarray/qPCR	different tissues and cultivars (Drakkar *vs*. Belinka)	secondary metabolism-associated genes highly represented in stems; 3 FLAs, laccases overexpressed in core tissue; 3 FLAs overexpressed in outer stem tissue; 6 LTPs, lipid/wax metabolism and photosynthesis-associated genes overexpressed in outer tissue; cultivars differ in cell wall- and biotic stress response-related genes	[84]
***L. usitatissimum***	cDNA library	bark	21 cell wall-related ESTs and C2H2 transcription factors identified	[85]
***L. usitatissimum***	lignomics/microarray	inner and outer stem tissues	hypolignification associated with low abundance of monolignol biosynthetic genes, 81 phenolic compounds found, 65 identified for the first time, lignan-associated genes abundant in inner tissues	[86]
***L. usitatissimum***	miRNA	bottom leaves, stem, leafless apex	20 miRNA identified, which could regulate cell wall metabolism	[87]
***L. usitatissimum***	Cell wall proteome, 1D-PAGE, MALDI-TOF	isolated fibres	List of potential cell wall proteins of flax fibres	[88]
***L. usitatissimum***	Cell wall proteome, LC-MS/MS	Cortical tissue, cell wall protein enrichment	Identification of > 150 predicted cell wall proteins, major group acting on sugars and glycoproteins	[89]
***B. nivea***	Illumina paired-end sequencing	xylem, shoot, leaves, bark from seedlings, 30- and 60-days old plants	51 genes of the *CesA* superfamily identified, 36 showing high expression in bark	[90]

List of abbreviations used: *CesA*s: cellulose synthase; XTH: xyloglucan endotransglycosylase/hydrolase; GT: glycosyltransferase; GH: glycosylhydrolase; PRPs: proline-rich proteins; AGPs: arabinogalactan proteins; PP: pentose phosphate; AAA: aromatic aminoacids; FLAs: fasciclin-like arabinogalactan proteins; LTP: lipid-transfer protein.

**Table 2:** Summary of the main physical parameters of fibres from fibre crops (adapted from [116]). MFA stands for microfibril angle.

Plant	Fibre type	Length (mm)	Diameter (μm)	MFA
Hemp	Bast	5–60	20–40	4°
Hemp	Hurd	0.2–0.6	10–30	0°–10° in S2 layer; 70°–90° in S1 layer
Flax	Bast	2–40	20–23	10°
Jute	Bast	2–3	16	8°
Ramie	Bast	40–150	30	8°
Sisal	Leaf	2–4	20	20°

## References

[1] Marques G., Rencoret J., Gutiérrez A., del Río J.C. (2010). Evaluation of the chemical composition of different non-woody plant fibers used for pulp and paper manufacturing. Open Agric. J.

[2] Benning C., Pichersky E. (2008). Harnessing plant biomass for biofuels and biomaterials. Plant J.

[3] Del Río J.C., Rencoret J., Gutiérrez A., Nieto L., Jiménez-Barbero J., Martínez Á.T. (2011). Structural characterization of guaiacyl-rich lignins in flax (*Linum usitatissimum*) fibers and shives. J. Agric. Food Chem..

[4] McNutt J.A., Haegglom R., Raemoe K (1992). The Global Fiber Resource Picture. Wood Product Demand and the Environment.

[5] Philippou L., Karastergiou S Lignocellulosic Materials From Annual Plants and Agricultural Residues As Raw Materials For Composite Building Materials.

[6] Reddy N., Yang Y. (2005). Biofibers from agricultural byproducts for industrial applications. Trends Biotechnol.

[7] Davies G.C., Bruce D.M. (1998). Effect of environmental relative humidity and damage on the tensile properties of flax and nettle fibers. Textil. Res. J.

[8] Fathima M., Balasubramanian A. (2006). Effect of plant growth regulators on the quality of bast fibres in *Abelmoschus esculentus* (Linn.) Moench. Acta Bot. Croat.

[9] Gorshkova T.A., Gurjanov O.P., Mikshina P.V., Ibragimova N.N., Mokshina N.E., Salnikov V.V., Ageeva M.V., Amenitskii S.I., Chernova T.E., Chemikosova S.B. (2010). Specific type of secondary cell wall formed by plant fibers. Russ. J. Plant Physiol.

[10] Lev-Yadun S. (2010). Plant fibers: Initiation, growth, model plants, and open questions. Russ. J. Plant Physiol.

[11] Gorshkova T., Brutch N., Chabbert B., Deyholos M., Hayashi T., Lev-Yadun S., Mellerowicz E.J., Morvan C., Neutelings G., Pilate G. (2012). Plant fiber formation: State of the art, recent and expected progress, and open questions. Crit. Rev. Plant Sci.

[12] Ko J.H., Yang S.H., Park A.H., Lerouxel O., Han K.H. (2007). ANAC012, a member of the plant–specific NAC transcription factor family, negatively regulates xylary fiber development in *Arabidopsis thaliana*. Plant J.

[13] Mitsuda N., Iwase A., Yamamoto H., Yoshida M., Seki M., Shinozaki K., Ohme-Takagi M. (2007). NAC transcription factors, NST1 and NST3, are key regulators of the formation of secondary walls in woody tissues of *Arabidopsis*. Plant Cell.

[14] Zhong R., Richardson E.A., Ye Z.H. (2007). The MYB46 transcription factor is a direct target of SND1 and regulates secondary wall biosynthesis in *Arabidopsis*. Plant Cell.

[15] Ko J.H., Kim W.C., Han K.H. (2009). Ectopic expression of MYB46 identifies transcriptional regulatory genes involved in secondary wall biosynthesis in *Arabidopsis*. Plant J.

[16] Bhargava A., Mansfield S.D., Hall H.C., Douglas C.J., Ellis B.E. (2010). MYB75 functions in regulation of secondary cell wall formation in the *Arabidopsis* inflorescence stem. Plant Physiol.

[17] Demura T., Ye Z.H. (2010). Regulation of plant biomass production. Curr. Opin. Plant Biol.

[18] Ohashi–Ito K., Oda Y., Fukuda H. (2010). *Arabidopsis* VASCULAR-RELATED NAC-DOMAIN6 directly regulates the genes that govern programmed cell death and secondary wall formation during xylem differentiation. Plant Cell.

[19] Zhong R., Lee C., Ye Z.H. (2010). Global analysis of direct targets of secondary wall NAC master switches in *Arabidopsis*. Mol. Plant.

[20] Yamaguchi M., Goué N., Igarashi H., Ohtani M., Nakano Y., Mortimer J.C., Nishikubo N., Kubo M., Katayama Y., Kakegawa K. (2010). VASCULAR-RELATED NAC-DOMAIN6 and VASCULAR-RELATED NAC-DOMAIN7 effectively induce transdifferentiation into xylem vessel elements under control of an induction system. Plant Physiol.

[21] Yamaguchi M., Mitsuda N., Ohtani M., Ohme-Takagi M., Kato K., Demura T. (2011). VASCULAR-RELATED NAC-DOMAIN7 directly regulates the expression of a broad range of genes for xylem vessel formation. Plant J.

[22] Wang H., Zhao Q., Chen F., Wang M., Dixon R.A. (2011). NAC domain function and transcriptional control of a secondary cell wall master switch. Plant J.

[23] Li E., Wang S., Liu Y., Chen J.G., Douglas C.J. (2011). OVATE FAMILY PROTEIN4 (OFP4) interaction with KNAT7 regulates secondary cell wall formation in *Arabidopsis thaliana*. Plant J.

[24] Kim W.C., Ko J.H., Han K.H. (2012). Identification of a *cis*-acting regulatory motif recognized by MYB46, a master transcriptional regulator of secondary wall biosynthesis. Plant Mol. Biol.

[25] Li E., Bhargava A., Qiang W., Friedmann M.C., Forneris N., Savidge R.A., Johnson L.A., Mansfield S.D., Ellis B.E., Douglas C.J. (2012). The Class II KNOX gene KNAT7 negatively regulates secondary wall formation in *Arabidopsis* and is functionally conserved in *Populus*. New Phytol.

[26] Bhargava A., Ahad A., Wang S., Mansfield S.D., Haughn G.W., Douglas C.J., Ellis B.E. (2013). The interacting MYB75 and KNAT7 transcription factors modulate secondary cell wall deposition both in stems and seed coat in *Arabidopsis*. Planta.

[27] Williamson R.E., Burn J.E., Birch R., Baskin T.I., Arioli T., Betzner A.S., Cork A. (2001). Morphology of rsw1, a cellulose-deficient mutant of *Arabidopsis thaliana*. Protoplasma.

[28] Burn J.E., Hurley U.A., Birch R.J., Arioli T., Cork A., Williamson R.E. (2002). The cellulose-deficient *Arabidopsis* mutant rsw3 is defective in a gene encoding a putative glucosidase II, an enzyme processing *N*-glycans during ER quality control. Plant J.

[29] Zhong R., Morrison W.H., Freshour G.D., Hahn M.G., Ye Z.H. (2003). Expression of a mutant form of cellulose synthase AtCesA7 causes dominant negative effect on cellulose biosynthesis. Plant Physiol.

[30] Szyjanowicz P.M., McKinnon I., Taylor N.G., Gardiner J., Jarvis M.C., Turner S.R. (2004). The irregular xylem 2 mutant is an allele of korrigan that affects the secondary cell wall of *Arabidopsis thaliana*. Plant J.

[31] Chen Z., Hong X., Zhang H., Wang Y., Li X., Zhu J.K., Gong Z. (2005). Disruption of the cellulose synthase gene, AtCesA8/IRX1, enhances drought and osmotic stress tolerance in *Arabidopsis*. Plant J.

[32] MacKinnon I.M., Sturcová A., Sugimoto-Shirasu K., His I, McCann M.C., Jarvis M.C. (2006). Cell-wall structure and anisotropy in procuste, a cellulose synthase mutant of *Arabidopsis thaliana*. Planta.

[33] Chu Z., Chen H., Zhang Y., Zhang Z., Zheng N., Yin B., Yan H., Zhu L., Zhao X., Yuan M. (2007). Knockout of the AtCESA2 gene affects microtubule orientation and causes abnormal cell expansion in *Arabidopsis*. Plant Physiol.

[34] Persson S., Caffall K.H., Freshour G., Hilley M.T., Bauer S., Poindexter P., Hahn M.G., Mohnen D., Somerville C. (2007). The *Arabidopsis* irregular xylem8 mutant is deficient in glucuronoxylan and homogalacturonan, which are essential for secondary cell wall integrity. Plant Cell.

[35] Bringmann M., Li E., Sampathkumar A., Kocabek T., Hauser M.T., Persson S. (2012). POM-POM2/cellulose synthase interacting1 is essential for the functional association of cellulose synthase and microtubules in *Arabidopsis*. Plant Cell.

[36] Sánchez-Rodríguez C., Bauer S., Hématy K., Saxe F., Ibáñez A.B., Vodermaier V., Konlechner C., Sampathkumar A., Rüggeberg M., Aichinger E. (2012). Chitinase-like1/pom-pom1 and its homolog CTL2 are glucan-interacting proteins important for cellulose biosynthesis in *Arabidopsis*. Plant Cell.

[37] Rubio-Díaz S., Pérez-Pérez J.M., González-Bayón R., Muñoz-Viana R., Borrega N., Mouille G., Hernández-Romero D., Robles P., Höfte H., Ponce M.R. (2012). Cell expansion-mediated organ growth is affected by mutations in three EXIGUA genes. PLoS One.

[38] Wyatt S.E., Sederoff R., Flaishman M.A., Lev-Yadun S. (2010). *Arabidopsis thaliana* as a model for gelatinous fiber formation. Russ. J. Plant Physiol.

[39] Rajangam A.S., Kumar M., Aspeborg H., Guerriero G., Arvestad L., Pansri P., Brown C.J., Hober S., Blomqvist K., Divne C. (2008). MAP20, a microtubule-associated protein in the secondary cell walls of hybrid aspen, is a target of the cellulose synthesis inhibitor 2,6-dichlorobenzonitrile. Plant Physiol.

[40] Winzell A., Aspeborg H., Wang Y., Ezcurra I. (2010). Conserved CA-rich motifs in gene promoters of Pt x tMYB021-responsive secondary cell wall carbohydrate-active enzymes in *Populus*. Biochem. Biophys. Res. Commun.

[41] Grant E.H., Fujino T., Beers E.P., Brunner A.M. (2010). Characterization of NAC domain transcription factors implicated in control of vascular cell differentiation in *Arabidopsis* and *Populus*. Planta.

[42] Zhong R., McCarthy R.L., Lee C., Ye Z.H. (2011). Dissection of the transcriptional program regulating secondary wall biosynthesis during wood formation in poplar. Plant Physiol.

[43] Yang X., Ye C.Y., Bisaria A., Tuskan G.A., Kalluri U.C. (2011). Identification of candidate genes in *Arabidopsis* and *Populus* cell wall biosynthesis using text-mining, co-expression network analysis and comparative genomics. Plant Sci.

[44] Li Q., Lin Y.C., Sun Y.H., Song J., Chen H., Zhang X.H., Sederoff R.R., Chiang V.L. (2012). Splice variant of the SND1 transcription factor is a dominant negative of SND1 members and their regulation in *Populus trichocarpa*. Proc. Natl. Acad. Sci. USA.

[45] Tian X., Xie J., Zhao Y., Lu H., Liu S., Qu L., Li J., Gai Y., Jiang X. (2013). Sense-, antisense- and RNAi-4CL1 regulate soluble phenolic acids, cell wall components and growth in transgenic *Populus tomentosa* Carr. Plant Physiol. Biochem.

[46] Tuskan G.A., Difazio S., Jansson S., Bohlmann J., Grigoriev I., Hellsten U., Putnam N., Ralph S., Rombauts S., Salamov A. (2006). The genome of black cottonwood, *Populus trichocarpa* (Torr. & Gray). Science.

[47] Ralph J., Lapierre C., Marita J.M., Kim H., Lu F., Hatfield R.D., Ralph S., Chapple C., Franke R., Hemm M.R. (2001). Elucidation of new structures in lignins of CAD- and COMT-deficient plants by NMR. Phytochemistry.

[48] Tesfaye M., Yang S.S., Lamb J.F.S., Jung H-J.G., Samac D.A., Vance C.P., Gronwald J.W., VandenBosch K.A. (2009). *Medicago truncatula* as a model for dicot cell wall development. Bioenergy Res..

[49] Zhao Q., Gallego-Giraldo L., Wang H., Zeng Y., Ding S.Y., Chen F., Dixon R.A. (2010). An NAC transcription factor orchestrates multiple features of cell wall development in *Medicago truncatula*. Plant J.

[50] Yang S.S., Tu Z.J., Cheung F., Xu W.W., Lamb J.F., Jung H.J., Vance C.P., Gronwald J.W. (2011). Using RNA-Seq for gene identification, polymorphism detection and transcript profiling in two alfalfa genotypes with divergent cell wall composition in stems. BMC Genomics.

[51] Lee Y., Chen F., Gallego-Giraldo L., Dixon R.A., Voit E.O. (2011). Integrative analysis of transgenic alfalfa (*Medicago sativa* L.) suggests new metabolic control mechanisms for monolignol biosynthesis. PLoS Comput. Biol.

[52] Van Bakel H., Stout J.M., Cote A.G., Tallon C.M., Sharpe A.G., Hughes T.R., Page J.E. (2011). The draft genome and transcriptome of *Cannabis sativa*. Genome Biol.

[53] Wang Z., Hobson N., Galindo L., Zhu S., Shi D., McDill J., Yang L., Hawkins S., Neutelings G., Datla R. (2012). The genome of flax (*Linum usitatissimum*) assembled *de novo* from short shotgun sequence reads. Plant J.

[54] Bothwell J.H. (2006). The long past of systems biology. New Phytol.

[55] Keurentjes J.J., Angenent G.C., Dicke M., Dos Santos V.A., Molenaar J., van der Putten W.H., de Ruiter P.C., Struik P.C., Thomma B.P. (2011). Redefining plant systems biology: From cell to ecosystem. Trends Plant Sci.

[56] Fraser C.M., Chapple C. (2011). The phenylpropanoid pathway in *Arabidopsis*. Arabidopsis Book.

[57] Winkel-Shirley B. (2001). Flavonoid biosynthesis. A colorful model for genetics, biochemistry, cell biology, and biotechnology. Plant Physiol.

[58] Millar D.J., Long M., Donovan G., Fraser P.D., Boudet A.M., Danoun S., Bramley P.M., Bolwell G.P. (2007). Introduction of sense constructs of cinnamate 4-hydroxylase (CYP73A24) in transgenic tomato plants shows opposite effects on flux into stem lignin and fruit flavonoids. Phytochemistry.

[59] Bonawitz N.D., Chapple C. (2010). The genetics of lignin biosynthesis: Connecting genotype to phenotype. Annu. Rev. Genet.

[60] Vanholme R., Morreel K., Darrah C., Oyarce P., Grabber J.H., Ralph J., Boerjan W. (2012). Metabolic engineering of novel lignin in biomass crops. New Phytol.

[61] Song L., Siguier B., Dumon C., Bozonnet S., O’Donohue M.J. (2012). Engineering better biomass-degrading ability into a GH11 xylanase using a directed evolution strategy. Biotechnol. Biofuels.

[62] Petersen P.D., Lau J., Ebert B., Yang F., Verhertbruggen Y., Kim J.S., Varanasi P., Suttangkakul A., Auer M., Loqué D. (2012). Engineering of plants with improved properties as biofuels feedstocks by vessel-specific complementation of xylan biosynthesis mutants. Biotechnol. Biofuels.

[63] Bonawitz N.D., Chapple C. (2013). Can genetic engineering of lignin deposition be accomplished without an unacceptable yield penalty?. Curr. Opin. Biotechnol.

[64] Chen F., Srinivasa Reddy M.S., Temple S., Jackson L., Shadle G., Dixon R.A. (2006). Multi-site genetic modulation of monolignol biosynthesis suggests new routes for formation of syringyl lignin and wall-bound ferulic acid in alfalfa (*Medicago sativa* L.). Plant J.

[65] Shadle G., Chen F., Srinivasa Reddy M.S., Jackson L., Nakashima J., Dixon R.A. (2007). Down-regulation of hydroxycinnamoyl CoA: Shikimate hydroxycinnamoyl transferase in transgenic alfalfa affects lignification, development and forage quality. Phytochemistry.

[66] Nakashima J., Chen F., Jackson L., Shadle G., Dixon R.A. (2008). Multi-site genetic modification of monolignol biosynthesis in alfalfa (*Medicago sativa*): Effects on lignin composition in specific cell types. New Phytol.

[67] Xu B., Escamilla-Treviño L.L., Sathitsuksanoh N., Shen Z., Shen H., Zhang Y.H., Dixon R.A., Zhao B. (2011). Silencing of 4-coumarate:coenzyme A ligase in switchgrass leads to reduced lignin content and improved fermentable sugar yields for biofuel production. New Phytol.

[68] Dien B.S., Miller D.J., Hector R.E., Dixon R.A., Chen F., McCaslin M., Reisen P., Sarath G., Cotta M.A. (2011). Enhancing alfalfa conversion efficiencies for sugar recovery and ethanol production by altering lignin composition. Bioresour. Technol.

[69] Ambavaram M.M., Krishnan A., Trijatmiko K.R., Pereira A. (2011). Coordinated activation of cellulose and repression of lignin biosynthesis pathways in rice. Plant Physiol.

[70] Fursova O., Pogorelko G., Zabotina O.A. (2012). An efficient method for transient gene expression in monocots applied to modify the *Brachypodium distachyon* cell wall. Ann Bot.

[71] Jung J.H., Vermerris W., Gallo M., Fedenko J.R., Erickson J.E., Altpeter F (2013). RNA interference suppression of lignin biosynthesis increases fermentable sugar yields for biofuel production from field-grown sugarcane. Plant Biotechnol. J..

[72] Grabber J.H., Schatz P.F., Kim H., Lu F., Ralph J. (2010). Identifying new lignin bioengineering targets: 1. Monolignol-substitute impacts on lignin formation and cell wall fermentability. BMC Plant Biol.

[73] Zhang K., Bhuiya M.W., Pazo J.R., Miao Y., Kim H., Ralph J., Liu C.J. (2012). An engineered monolignol 4-*O*-methyltransferase depresses lignin biosynthesis and confers novel metabolic capability in *Arabidopsis*. Plant Cell.

[74] Elumalai S., Tobimatsu Y., Grabber J.H., Pan X., Ralph J. (2012). Epigallocatechin gallate incorporation into lignin enhances the alkaline delignification and enzymatic saccharification of cell walls. Biotechnol. Biofuels.

[75] Gorshkova T.A., Salnikov V.V., Pogodina N.M., Chemikosova S.B., Yablokova E.V., Ulanov A.V., Ageeva M.V., van Dam J.E.G., Lozovaya V.V. (2000). Composition and distribution of cell wall phenolic compounds in flax (*Linum usitatissimum* L.) stem tissues. Ann. Bot.

[76] Day A., Ruel K., Neutelings G., Crônier D., David H., Hawkins S., Chabbert B. (2005). Lignification in the flax stem: Evidence for an unusual lignin in bast fibers. Planta.

[77] Day A., Addi M., Kim W., David H., Bert F., Mesnage P., Rolando C., Chabbert B., Neutelings G., Hawkins S. (2005). ESTs from the fibre-bearing stem tissues of flax (*Linum usitatissimum* L.): Expression analyses of sequences related to cell wall development. Plant Biol.

[78] Crônier D., Monties B., Chabbert B. (2005). Structure and chemical composition of bast fibers isolated from developing hemp stem. J. Agric. Food Chem.

[79] De Pauw M.A., Vidmar J.J., Collins J., Bennett R.A., Deyholos M.K. (2007). Microarray analysis of bast fibre producing tissue of *Cannabis sativa* identifies transcripts associated with conserved and specialised processes of secondary wall development. Func. Plant Biol.

[80] Roach M.J., Deyholos M.K. (2007). Microarray analysis of flax (*Linum usitatissimum* L.) stems identifies transcripts enriched in fibre-bearing phloem tissues. Mol. Genet. Genomics.

[81] Van den Broeck H.C., Maliepaard C., Ebskam M.J.M., Toonen M.A.J., Koops A.J. (2008). Differential expression of genes involved in C1 metabolism and lignin biosynthesis in wooden core and bast tissues of fibre hemp (*Cannabis sativa* L.). Plant Sci.

[82] Hotte N.S., Deyholos M.K. (2008). A flax fibre proteome: Identification of proteins enriched in bast fibres. BMC Plant Biol.

[83] Roach M.J., Deyholos M.K. (2008). Microarray analysis of developing flax hypocotyls identifies novel transcripts correlated with specific stages of phloem fibre differentiation. Ann. Bot.

[84] Fénart S., Ndong Y.P., Duarte J., Rivière N., Wilmer J., van Wuytswinkel O., Lucau A., Cariou E., Neutelings G., Gutierrez L. (2010). Development and validation of a flax (*Linum usitatissimum* L.) gene expression oligo microarray. BMC Genomics.

[85] Long S.H., Deng X., Wang Y.F., Li X., Qiao R.Q., Qiu C.S., Guo Y., Hao D.M., Jia W.Q., Chen X.B. (2012). Analysis of 2.297 expressed sequence tags (ESTs) from a cDNA library of flax (*Linum ustitatissimum* L.) bark tissue. Mol. Biol. Rep.

[86] Huis R., Morreel K., Fliniaux O., Lucau-Danila A., Fénart S., Grec S., Neutelings G., Chabbert B., Mesnard F., Boerjan W. (2012). Natural hypolignification is associated with extensive oligolignol accumulation in flax stems. Plant Physiol.

[87] Neutelings G., Fénart S., Lucau-Danila A., Hawkins S. (2012). Identification and characterization of miRNAs and their potential targets in flax. J. Plant Physiol.

[88] Ibragimova N.N., Mokshina N.E., Gorshkova T.A. (2012). Cell wall proteins of flax phloem fibers. Russ. J. Bioorg. Chem.

[89] Day A., Fénart S., Neutelings G., Hawkins S., Rolando C., Tokarski C. (2013). Identification of cell wall proteins in the flax (*Linum usitatissimum*) stem. Proteomics.

[90] Liu T., Zhu S., Tang Q., Chen P., Yu Y., Tang S. (2013). *De novo* assembly and characterization of transcriptome using Illumina paired-end sequencing and identification of *CesA* gene in ramie (*Boehmeria nivea* L. *Gaud*). BMC Genomics.

[91] Achyuthan K.E., Achyuthan A.M., Adams P.D., Dirk S.M., Harper J.C., Simmons B.A., Singh A.K. (2010). Supramolecular self-assembled chaos: Polyphenolic lignin’s barrier to cost-effective lignocellulosic biofuels. Molecules.

[92] Gandolfi S., Ottolina G., Riva S., Predrocchi Fantoni G., Patel I. (2013). Complete chemical analysis of carmagnola hemp hurds and structural features of its components. BioResources.

[93] Del Río J.C., Rencoret J., Prinsen P., Martinez A.T., Ralph J., Gutierrez A. (2012). Structural characterization of wheat straw lignin as revealed by analytical pyrolysis, 2D-NMR, and reductive cleavage methods. J. Agric. Food Chem.

[94] Amthor J.S. (2003). Efficiency of lignin biosynthesis: A quantitative analysis. Ann. Bot.

[95] Casler M.D., Buxton D.R., Vogel K.P. (2002). Genetic modification of lignin concentration affects fitness of perennial herbaceous plants. Theor. Appl. Genet.

[96] Moura J.C., Bonine C.A., de Oliveira F.V.J., Dornelas M.C., Mazzafera P. (2010). Abiotic and biotic stresses and changes in the lignin content and composition in plants. J. Integr. Plant Biol.

[97] Hamberger B., Hahlbrock K. (2004). The 4-coumarate:CoA ligase gene family in *Arabidopsis thaliana* comprises one rare, sinapate-activating and three commonly occurring isoenzymes. Proc. Natl. Acad. Sci. USA.

[98] Costa M.A., Bedgar D.L., Moinuddin S.G., Kim K.W., Cardenas C.L., Cochrane F.C., Shockey J.M., Helms G.L., Amakura Y., Takahashi H. (2005). Characterization *in vitro* and *in vivo* of the putative multigene 4-coumarate:CoA ligase network in *Arabidopsis*: Syringyl lignin and sinapate/sinapyl alcohol derivative formation. Phytochemistry.

[99] Lu S., Zhou Y., Li L., Chiang V.L. (2006). Distinct roles of cinnamate 4-hydroxylase genes in *Populus*. Plant Cell Physiol.

[100] Soltani B.M., Ehlting J., Hamberger B., Douglas C.J. (2006). Multiple *cis*-regulatory elements regulate distinct and complex patterns of developmental and wound-induced expression of *Arabidopsis thaliana* 4CL gene family members. Planta.

[101] Huang J., Gu M., Lai Z., Fan B., Shi K., Zhou Y.H., Yu J.Q., Chen Z. (2010). Functional analysis of the *Arabidopsis* PAL gene family in plant growth, development, and response to environmental stress. Plant Physiol.

[102] Rasmussen S., Dixon R.A. (1999). Transgene-mediated and elicitor-induced perturbation of metabolic channeling at the entry point into the phenylpropanoid pathway. Plant Cell.

[103] Achnine L., Blancaflor E.B., Rasmussen S., Dixon R.A. (2004). Colocalization of L-phenylalanine ammonia-lyase and cinnamate 4-hydroxylase for metabolic channeling in phenylpropanoid biosynthesis. Plant Cell.

[104] Bassard J.E., Richert L., Geerinck J., Renault H., Duval F., Ullmann P., Schmitt M., Meyer E., Mutterer J., Boerjan W. (2012). Protein-protein and protein-membrane associations in the lignin pathway. Plant Cell.

[105] Jones L., Ennos A.R., Turner S.R. (2001). Cloning and characterization of irregular xylem4 (irx4): A severely lignin-deficient mutant of *Arabidopsis*. Plant J.

[106] Leplé J.C., Dauwe R., Morreel K., Storme V., Lapierre C., Pollet B., Naumann A., Kang K.Y., Kim H., Ruel K. (2007). Downregulation of cinnamoyl-coenzyme A reductase in poplar: Multiple-level phenotyping reveals effects on cell wall polymer metabolism and structure. Plant Cell.

[107] Zhou R, Jackson L, Shadle G, Nakashima J, Temple S, Chen F, Dixon R.A. (2010). Distinct cinnamoyl CoA reductases involved in parallel routes to lignin in *Medicago truncatula*. Proc. Natl. Acad. Sci. USA.

[108] Kaneda M., Schuetz M., Lin B.S., Chanis C., Hamberger B., Western T.L., Ehlting J., Samuels A.L. (2011). ABC transporters coordinately expressed during lignification of *Arabidopsis* stems include a set of ABCBs associated with auxin transport. J. Exp. Bot.

[109] Alejandro S., Lee Y., Tohge T., Sudre D., Osorio S., Park J., Bovet L., Lee Y., Geldner N., Fernie A.R. (2012). AtABCG29 is a monolignol transporter involved in lignin biosynthesis. Curr. Biol.

[110] Liu C.J. (2012). Deciphering the enigma of lignification: Precursor transport, oxidation, and the topochemistry of lignin assembly. Mol. Plant.

[111] Neutelings G. (2011). Lignin variability in plant cell walls: Contribution of new models. Plant Sci.

[112] Cabané M., Pireaux J.C., Léger E., Weber E., Dizengremel P., Pollet B., Lapierre C. (2004). Condensed lignins are synthesized in poplar leaves exposed to ozone. Plant Physiol.

[113] Pejic B.M., Kostic M.M., Skundric P.D., Praskalo J.Z. (2008). The effects of hemicelluloses and lignin removal on water uptake behavior of hemp fibers. Bioresour. Technol.

[114] Mikshina P.V., Chemikosova S.B., Mokshina N.E., Ibragimova N.N., Gorshkova T.A. (2009). Free galactose and galactosidase activity in the course of flax fiber development. Russ. J. Plant Physiol.

[115] Hobson N., Roach M.J., Deyholos M.K. (2010). Gene expression in tension wood and bast fibres. Russ. J. Plant Physiol..

[116] Thygesen A (2005). Properties of Hemp Fibre Polymer Composites—An Optimisation of Fibre Properties Using Novel Defibration Methods and Fibre Characterization. Ph.D. Thesis.

[117] Clair B., Alméras T., Yamamoto H., Okuyama T., Sugiyama J. (2006). Mechanical behavior of cellulose microfibrils in tension wood, in relation with maturation stress generation. Biophys. J.

[118] Mellerowicz E.J., Immerzeel P., Hayashi T. (2008). Xyloglucan: The molecular muscle of trees. Ann. Bot.

[119] Gorshkova T.A., Chemikosova S.B., Salnikov V.V., Pavlencheva N.V., Gurjanov O.P., Stolle-Smits T., van Dam J.E.G. (2004). Occurrence of cell-specific galactan is coinciding with bast fiber developmental transition in flax. Ind. Crops Prod.

[120] Mellerowicz E.J., Gorshkova T.A. (2012). Tensional stress generation in gelatinous fibres: A review and possible mechanism based on cell-wall structure and composition. J. Exp. Bot.

[121] Snegireva A.V., Ageeva M.V., Amenitskii S.I., Chernova T.E., Ebskamp M., Gorshkova T.A. (2010). Intrusive growth of sclerenchyma fibers. Russ. J. Plant Physiol.

[122] Gorshkova T.A., Sal’nikov V.V., Chemikosova S.B., Ageeva M.V., Pavlencheva N.V. (2003). The snap point: A transition point in *Linum usitatissimum* bast fiber development. Ind. Crops Prod.

[123] Ageeva M.V., Petrovská B., Kieft H., Sal’nikov V.V., Snegireva A.V., van Dam J.E., van Veenendaal W.L., Emons A.M., Gorshkova T.A., van Lammeren A.A. (2005). Intrusive growth of flax phloem fibers is of intercalary type. Planta.

[124] Gorshkova T., Morvan C. (2006). Secondary cell-wall assembly in flax phloem fibres: Role of galactans. Planta.

[125] Salnikov V.V., Ageeva M.V., Gorshkova T.A. (2008). Homofusion of Golgi secretory vesicles in flax phloem fibers during formation of the gelatinous secondary cell wall. Protoplasma.

[126] Fukushima A., Kusano M., Redestig H., Arita M., Saito K. (2009). Integrated omics approaches in plant systems biology. Curr. Opin. Chem. Biol.

[127] Weckwerth W. (2011). Green systems biology—From single genomes, proteomes and metabolomes to ecosystems research and biotechnology. J. Proteomics.

[128] García-Alcalde F., García-López F., Dopazo J., Conesa A. (2011). Paintomics: A web based tool for the joint visualization of transcriptomics and metabolomics data. Bioinformatics.

[129] Lin K., Kools H., de Groot P.J., Gavai A.K., Basnet R.K., Cheng F., Wu J., Wang X., Lommen A., Hooiveld G.J. (2011). MADMAX—Management and analysis database for multiple ~omics experiments. J. Integr. Bioinform.

[130] Syed M.H., Karpinets T.V., Parang M., Leuze M.R., Park B.H., Hyatt D., Brown S.D., Moulton S., Galloway M.D., Uberbacher E.C. (2012). BESC knowledgebase public portal. Bioinformatics.

[131] Sun X., Weckwerth W. (2012). COVAIN: A toolbox for uni- and multivariate statistics, time-series and correlation network analysis and inverse estimation of the differential Jacobian from metabolomics covariance data. Metabolomics.

[132] Fukushima A., Kusano M. (2013). Recent progress in the development of metabolome databases for plant systems biology. Front. Plant Sci.

[133] Liepe J., Filippi S., Komorowski M., Stumpf M.P. (2013). Maximizing the information content of experiments in systems biology. PLoS Comput. Biol.

[134] Chiaiese P., Ruotolo G., di Matteo A., de Santo Virzo A., de Marco A., Filippone E. (2011). Cloning and expression analysis of kenaf (*Hibiscus cannabinus* L.) major lignin and cellulose biosynthesis gene sequences and polymer quantification during plant development. Ind. Crops Prod.

[135] Jeong M.-J., Choi B.S., Bae D.W., Shin S.C., Park S.U., Lim H.-S., Kim J., Kim J.B., Cho B.-K., Bae H. (2012). Differential expression of kenaf phenylalanine ammonia-lyase (PAL) ortholog during developmental stages and in response to abiotic stresses. Plant Omics.

[136] Chowdhury E.M.D., Choi B.S., Park S.U., Lim H.-S., Bae H. (2012). Transcriptional analysis of hydroxycinnamoyl transferase (HCT) in various tissues of *Hibiscus cannabinus* in response to abiotic stress conditions. Plant Omics.

[137] Kumar S., You F.M., Cloutier S. (2012). Genome wide SNP discovery in flax through next generation sequencing of reduced representation libraries. BMC Genomics.

[138] Martin L.B., Fei Z., Giovannoni J.J., Rose J.K. (2013). Catalyzing plant science research with RNA-seq. Front. Plant Sci.

[139] Puzey J.R., Karger A., Axtell M., Kramer E.M. (2012). Deep annotation of *Populus trichocarpa* microRNAs from diverse tissue sets. PLoS One.

[140] Ong S.S., Wickneswari R. (2012). Characterization of microRNAs expressed during secondary wall biosynthesis in *Acacia mangium*. PLoS One.

[141] Lu S., Sun Y.H., Shi R., Clark C., Li L., Chiang V.L. (2005). Novel and mechanical stress-responsive microRNAs in *Populus trichocarpa* that are absent from *Arabidopsis*. Plant Cell.

[142] Fagerstedt K.V., Kukkola E.M., Koistinen V.V., Takahashi J., Marjamaa K. (2010). Cell wall lignin is polymerised by class III secretable plant peroxidases in Norway spruce. J. Integr. Plant Biol.

[143] Marjamaa K., Kukkola E.M., Fagerstedt K.V. (2009). The role of xylem class III peroxidases in lignification. J. Exp. Bot.

[144] Li X., Weng J.K., Chapple C. (2008). Improvement of biomass through lignin modification. Plant J.

[145] Raes J., Rohde A., Christensen J.H., Van de Peer Y., Boerjan W. (2003). Genome-wide characterization of the lignification toolbox in *Arabidopsis*. Plant Physiol.

